# Quantum Hall device data monitoring following encapsulating polymer deposition

**DOI:** 10.1016/j.dib.2018.08.121

**Published:** 2018-08-30

**Authors:** Albert F. Rigosi, Chieh-I Liu, Bi Yi Wu, Hsin-Yen Lee, Mattias Kruskopf, Yanfei Yang, Heather M. Hill, Jiuning Hu, Emily G. Bittle, Jan Obrzut, Angela R. Hight Walker, Randolph E. Elmquist, David B. Newell

**Affiliations:** a National Institute of Standards and Technology (NIST), Gaithersburg, MD 20899, United States; b Graduate Institute of Applied Physics, National Taiwan University, Taipei 10617, Taiwan; c Joint Quantum Institute, University of Maryland, College Park, MD 20742, United States; d Theiss Research, La Jolla, CA 92037, United States

## Abstract

The information provided in this data article will cover the growth parameters for monolayer, epitaxial graphene, as well as how to verify the layer homogeneity by confocal laser scanning and optical microscopy. The characterization of the subsequently fabricated quantum Hall device is shown for example cases during a series of environmental exposures. Quantum Hall data acquired from a CYTOP encapsulation is also provided. Data from Raman spectroscopy, atomic force microscopy, and other electrical property trends are shown. Lastly, quantum Hall effect data are presented from devices with deposited Parylene C films measuring 10.7 μm and 720 nm. All data are relevant for Rigosi et al. [1].

**Specifications table**

**Table:** 

Subject area	*Physics*
More specific subject area	*Condensed Matter, Quantum Hall Effect*
Type of data	*Table, images, graphs*
How data was acquired	*Confocal laser scanning microscope [Olympus LEXT OLS4100], optical microscope [Nikon MM400, DS Ri2 Camera], Janis Research cryostat and magnet system [model 8TM-TLSL-HE3–17], atomic force microscope [Asylum Cypher], Raman spectroscopy [Renishaw InVia]*
Data format	*Raw data is graphed*
Experimental factors	*Parylene and CYTOP deposition*
Experimental features	*Monitor quantum Hall effect parameters after environmental exposures*
Data source location	*National Institute of Standards and Technology (U.S. Department of Commerce), 100 Bureau Drive, Gaithersburg, MD 20899*
Data accessibility	*Data is with this article*
Related research article	*Albert F. Rigosi, Chieh-I Liu, Bi Yi Wu, Hsin-Yen Lee, Mattias Kruskopf, Yanfei Yang, Heather M. Hill, Jiuning Hu, Emily G. Bittle, Jan Obrzut, Angela R. Hight Walker, Randolph E. Elmquist, and David B. Newell. Examining epitaxial graphene surface conductance and quantum Hall device stability with Parylene passivation. Microelectronic Engineering (in press).*

**Value of the data**

• The data provided in this submission can be used to help other researchers gauge the level of electrical stability needed for a variety of two-dimensional materials, especially those whose properties may drift with time due to atmospheric doping.
• These data can serve as a guide to further research in Parylene encapsulation.
• Those conducting research with epitaxial graphene can use the images, AFM, and Raman provided as a reference guide to identifying the correct number of the grown graphitic layers and for layer numbers in other van der Waals materials.

## 1 Data

### 1.1 Characterization of epitaxial graphene quantum Hall devices

**Fig. 1:**
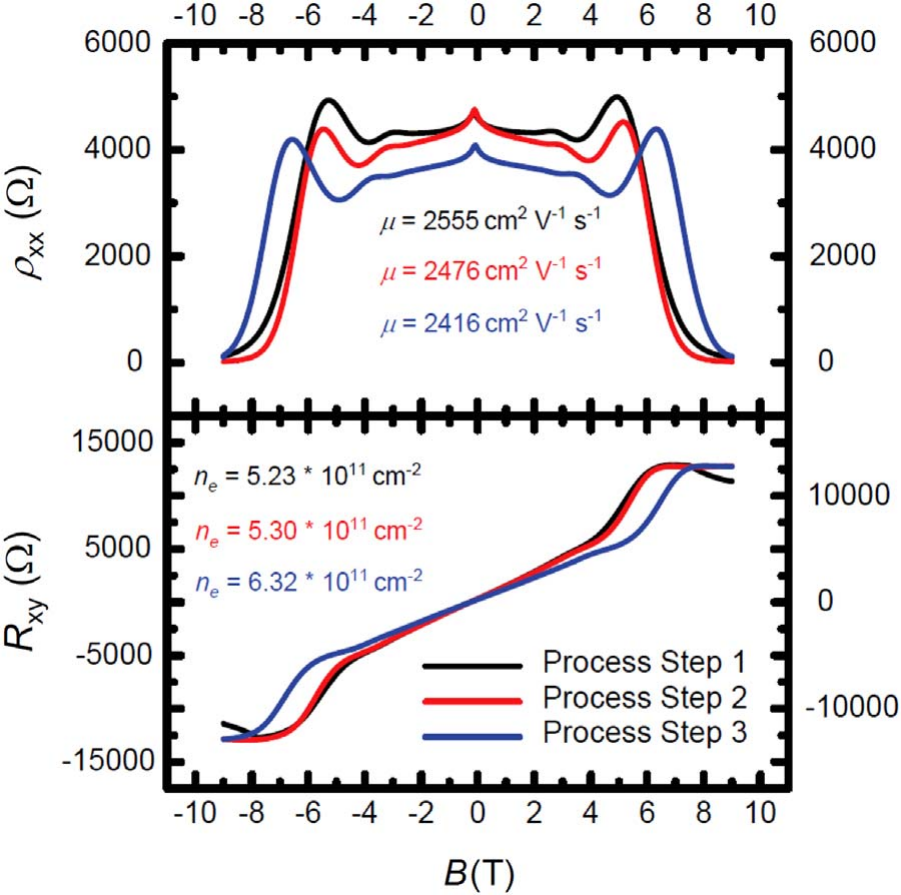
Quantum Hall measurements are shown here to demonstrate how the four parameters of interest change with the three example process steps.

After the growth and verification procedures described in the methods section, epitaxial graphene (EG) are fabricated into quantum Hall devices and characterized with a Janis Research cryostat and magnet system (model 8TM-TLSL-HE3–17).^∂^ Four relevant quantum Hall parameters are the Hall resistance (*R*_*xy*_), electron density (*n*_*e*_)*,* mobility (*μ*)*,* and longitudinal resistivity (*ρ*_*xx*_), and they are all measured and calculated ($n_{e}=\frac{1}{e\left(\frac{dR_{\mathrm{xy}}}{\mathrm{dB}}\right)}$ and $\mu =\frac{1}{en_{e}R_{\mathrm{xx}}\frac{W}{L}}$, where W and L are the width and length of the Hall device, respectively) as a function of up to nine process steps described in detail in Reference [1]. An example of how these parameters are monitored is shown in Fig. 1. The three example process steps are listed as such: A measurement on the four parameters is collected, followed by an exposure to a 60 °C and 85% relative humidity environment (using a Thermotron^∂^ environmental chamber), measurement collected, a repeated exposure to 60 °C and 85% relative humidity, measurement collected, and storage in air for two weeks, followed by a final measurement.

### 1.2 Polyperfluoro-butenylvinyl ether (CYTOP) encapsulation to attempt electrical stabilization of quantum Hall parameters

**Fig. 2:**
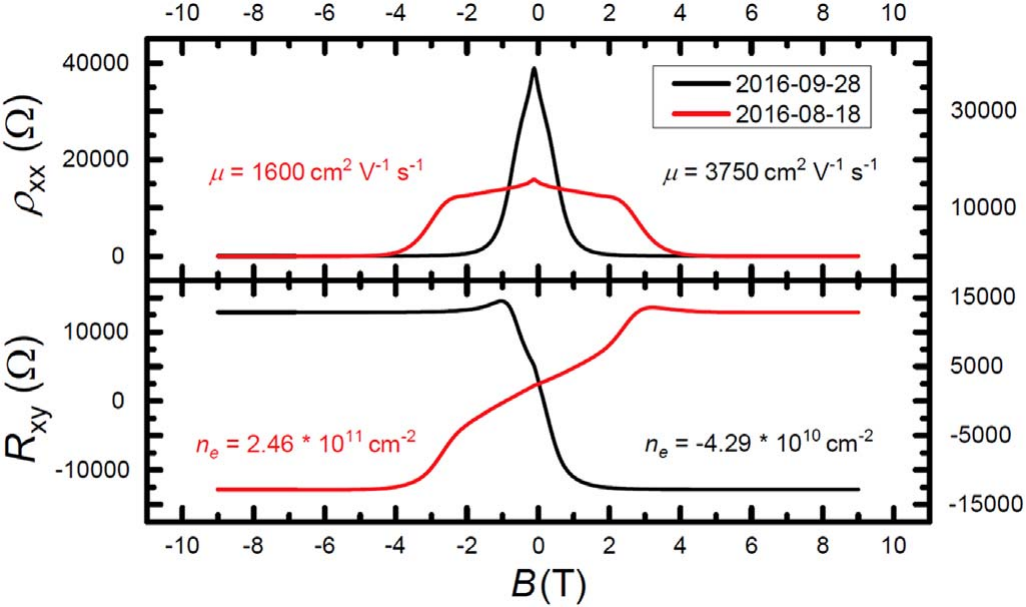
The device coated with CYTOP did not exhibit any signs of passivation, as shown by the longitudinal and Hall resistance measurements taken as a function of the magnetic field. After forty days of storage in ambient laboratory conditions, all four quantum Hall parameters modulated strongly.

When an EG device is encapsulated with CYTOP, the test for passivation capabilities is to store the device in ambient laboratory conditions for prolonged periods of time. The device was stored at 22 °C and 45% relative humidity for forty days. After the storing period, the usual four quantum Hall parameters were measured to characterize the electrical properties of the device. The corresponding data are shown in Fig. 2.

### 1.3 Raman spectroscopy, atomic force microscopy, and density-mobility relation data for an example epitaxial graphene quantum Hall device

**Fig. 3:**
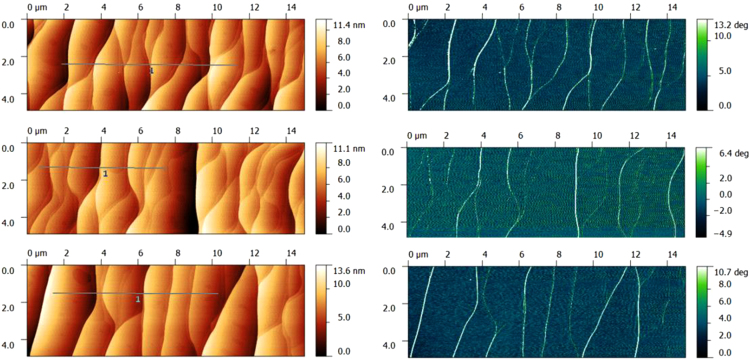
Three different example areas of EG, which eventually become devices or elongated areas for the microwave cavity, are inspected with an Asylum Cypher.^*∂*^ The topography is shown on the left side in golden color scale, while the phase is shown on the right with an aquamarine color scale. The edges of the SiC steps are clearly visible in these images, and on each terrace, the EG is uniform.

All EG samples were verified by optical microscopy before fabrication. Shortly after the growth, atomic force microscopy (AFM) was used to verify the general coverage of the EG, as seen in Fig. 3. An Asylum Cypher^*∂*^ was used to gather topographic and phase AFM images in tapping mode at 1 Hz, with image sizes being 15 µm by 5 µm.

**Fig. 4:**
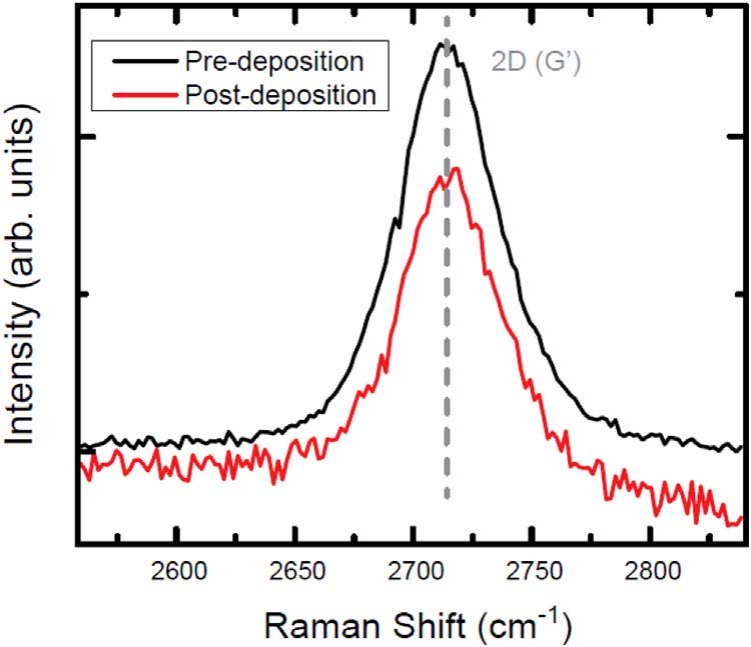
The Raman spectra above are used to verify that the EG is relatively unchanged. Following the Parylene deposition, an example device shows no change in position and a 5 cm^−1^ decrease in width of 2D (G′) peak at 2714 cm^−1^. The black and red curves are the Raman spectra before and after the deposition, respectively.

Raman spectra were collected after EG growth and device fabrication using a continuous-wave laser excitation at 632.8 nm in a commercial Renishaw InVia Raman^*∂*^ microscope. The purpose of the Raman was to verify that the EG was not defected. Spectral maps were acquired to ensure reproducibility of the EG Raman signals and were collected using a backscattering configuration with the following parameters: 5 by 3 raster-style grid of 20 µm steps, 1 μm spot size, 300 s acquisition time, 1.7 mW power, 50 × objective, and 1200 mm^−1^ grating. Some example Raman spectra showing the 2D (G′) peak are shown in Fig. 4.

**Fig. 5:**
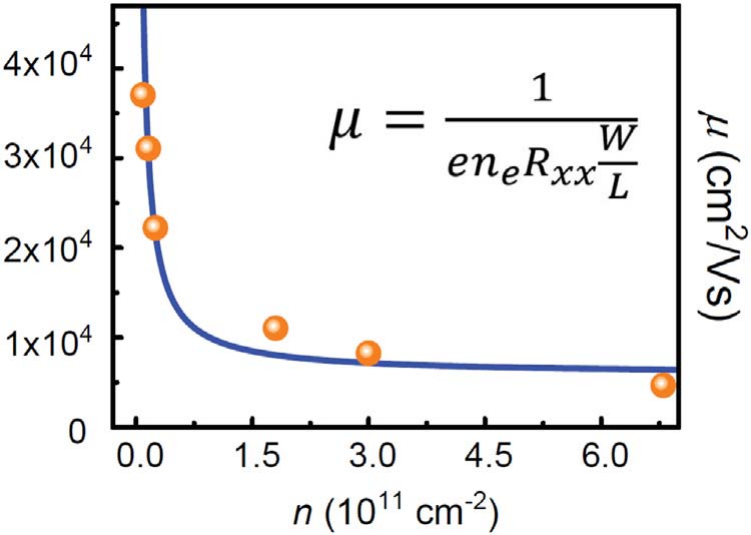
The relationship between mobility and carrier density is shown for one of the EG devices. Typically, when carrier densities are already higher than 1.5 × 10^11^ cm^−2^, drastic increases to the carrier density will not strongly modulate the mobility.

Lastly, an example device is tested for mapping out the relationship between carrier density and mobility, whose results are shown in Fig. 5.

### 1.4 Data indicating effects of thicker Parylene on quantum Hall parameters

**Fig. 6:**
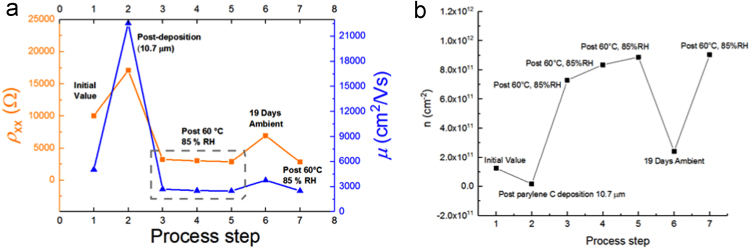
Parylene C was deposited with a total thickness of 10.7 μm. (a) The longitudinal resistivity and mobility (shown in orange and blue curves, respectively) of the device are tracked as a function of various processing steps to test the Parylene coating. The dotted gray box is meant to highlight that repeated exposures were performed. (b) The carrier density is monitored as well, showing a similar range of modulation as with the 720 nm Parylene thicknesses.

In one iteration of calibrating the Parylene deposition process, a 10.7 μm thickness was measured for one of the devices, which was tested to compare with the 720 nm thickness data presented in Ref. [1], [1] based on identical process steps. (Fig. 6).

### 1.5 Extra monitoring data

**Fig. 7:**
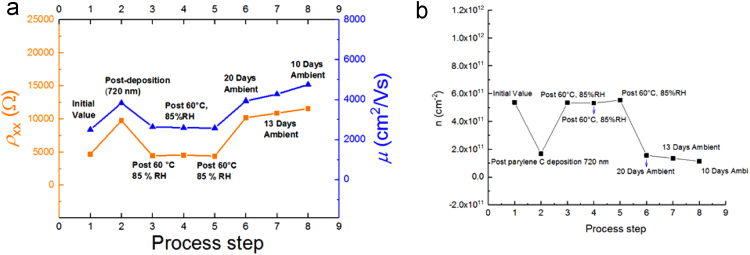
Parylene C was deposited with a total thickness of 720 nm. (a) Both the longitudinal resistivity and mobility are tracked as orange and blue curves, respectively, and labeled with the preceding exposure at each data point. (b) The carrier density is also monitored and plotted for the same process steps.

This section provides additional data for other devices that have been tested and exposed to the same environmental conditions as described in Ref. [1], [1]. Fig. 7 shows another device whose three electrical quantities were monitored as a function of process step.

## 2 Experimental design, materials, and methods

### 2.1 Epitaxial graphene growth and verification

The epitaxial graphene (EG) samples are grown on the silicon face of 4H-SiC(0001) semi-insulating substrates purchased from Cree, Inc.^∂^ The substrates have a miscut of about 0.10°. SiC substrates are submerged in a 5:1 diluted solution of hydrofluoric acid and deionized water, making an effective concentration of less than 10%. After rinsing with deionized water, substrates are placed on top of a polished pyrolytic graphite substrate from SPI Glass 22^∂^ with the SiC(0001) face resting against the graphite to promote homogeneous growth [2], [3]. Table 1 summarizes the growth parameters of several used samples:

**Table 1:** Various epitaxial growth conditions for all samples coated by Parylene. The red text indicates a sample tested for CYTOP encapsulation.

Sample ID	Ar gas flow (cm^3^/min)	Time at target (s)	Target Temperature (°C)	Target Std. Dev T (°C)
C7.1_J07_173	300	719	1750.27	0.74
C7.1_J15_173	300	719	1750.27	0.74
C7.3_T07_174	300	121	1900.46	1.03
C7.1_H07_183	300	718	1750.20	0.42
C7.8_H19_203	300	206	1900.43	0.69
C7.8_G05_206	300	186	1900.46	0.71
C7.8_G05_234	300	418	1900.58	1.34
C7.8_O19_246	300	296	1900.81	1.58
C7.8_H19_234	300	418	1900.58	1.34
C9.0_320_A	300	263	1900.86	1.67
C9.0_320_B	300	263	1900.86	1.67

**Fig. 8:**
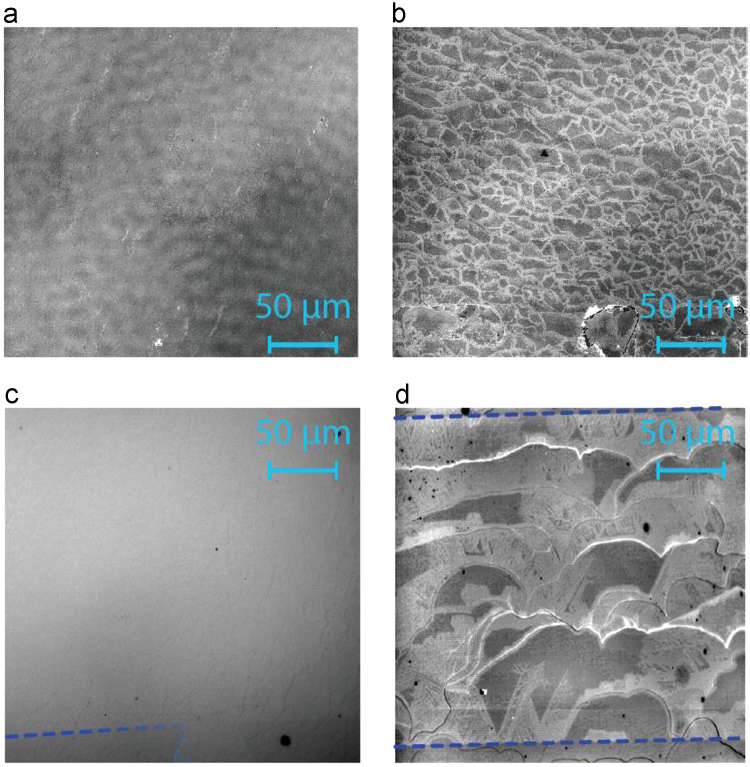
Examples of four different growths are shown. (a) CLSM image shows an example of one of the monolayer samples, with a sparse population of bilayer growth on terrace edges, as indicated by 10–20 μm long strips (and unit micron width) with brighter intensity. (b) An optical image second monolayer sample is shown with the graphene in most of the image. The boundary of the Hall device can be seen with a gradually disappearing, blue, dashed line to guide the eye. (c) An example of a sample *not* used for data collection is shown to provide a contrast in quality of graphene homogeneity. This CLSM image clarifies the difference between regions of bare SiC (or the buffer layer that precedes graphene growth) and the EG, which has only formed from the edges and has a lighter contrast than the darker SiC. (d) Another Hall device is optically imaged and shown as an example of a poorly-grown and *unused* device. With the dashed blue lines indicating the top and bottom boundaries of the device, the EG, of lighter contrast, has only grown on approximately half of the total region of the device. Furthermore, bilayers can be seen along terrace edges that span about 100 μm.

To demonstrate that monolayer graphene has been successfully grown, a combination of confocal laser scanning microscopy and optical imagining was utilized, as reported in previous work [4], [5]. Images are shown in Fig. 8 below and were acquired with a confocal laser scanning microscope (Olympus LEXT OLS4100)^∂^ and an optical microscope (Nikon MM400, DS Ri2 Camera).^∂^ All CLSM images have the contrast optimized by selecting the region in the light intensity histograms available in the LEXT^∂^ software containing at least 95.4% of the light (2σ). All optical images have the contrast optimized by selecting the region in the look up tables (LUTs) of the Nikon^∂^ software containing 99.6% of the light intensity (3σ) for each of the color channels (red, blue, and green).

### 2.2 Polyperfluoro-butenylvinyl ether (CYTOP) encapsulation

**Fig. 9:**
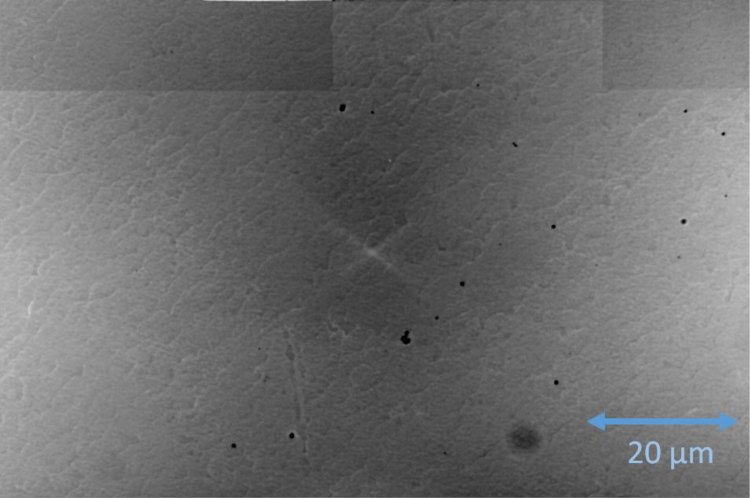
The fabricated device in the shape of a Hall bar is shown in one region. The lighter shade indicates the presence of graphene, while its texture is a result of the SiC steps formed beneath the graphene during the growth.

An additional polymer encapsulation material was used for comparing to the Parylene varieties. Polyperfluoro-butenylvinyl ether (CYTOP) was deposited on a graphene device by mixing a 1:1 solution of CYTOP CTX-809A and Solvent CT-SOLV180 by volume. The solution is spun onto the sample for 5 s at 500 revolutions per minute, and then for 40 s at 2000 revolutions per minute. To cure the solution on the surface of the chip, it was left at room temperature for 5 min, then at 50 °C for 40 min, 80 °C for 45 min, and 220 °C for 60 min. After the curing process, the sample was cooled on a hot plate for 20 min. This deposition yielded a 70 nm film. A filtered optical image of the device is shown in Fig. 9, while Fig. 2 shows the data from the encapsulated device.
